# Gamma-Aminobutyric Acid Levels in the Anterior Cingulate Cortex of Perimenopausal Women With Depression: A Magnetic Resonance Spectroscopy Study

**DOI:** 10.3389/fnins.2019.00785

**Published:** 2019-08-20

**Authors:** Dan Wang, Xuan Wang, Meng-Ting Luo, Hui Wang, Yue-Hua Li

**Affiliations:** ^1^Institute of Diagnostic and Interventional Radiology, Shanghai Sixth People’s Hospital, Shanghai Jiao Tong University, Shanghai, China; ^2^Department of Radiology, Henan Provincial People’s Hospital, Zhengzhou, China; ^3^Department of Otolaryngology Head and Neck Surgery, Shanghai Sixth People’s Hospital, Shanghai Jiao Tong University, Shanghai, China

**Keywords:** magnetic resonance spectroscopy, gamma-aminobutyric acid, postmenopausal, depression, anxiety, anterior cingulate cortex

## Abstract

**Objective:**

The anterior cingulate cortex (ACC) is associated with the processing of negative emotions. Gamma-aminobutyric acid (GABA) metabolism plays an important role in the pathogenesis of mental disorders. We aimed to determine the changes in GABA levels in the ACC of perimenopausal women with depression.

**Methods:**

We recruited 120 perimenopausal women, who were followed up for 18–24 months. After reaching menopause, the participants were divided into a control group (*n* = 71), an anxiety group (*n* = 30), and a depression group (*n* = 19). The participants were examined using proton magnetic resonance spectroscopy (MRS). TARQUIN software was used to calculate the GABA concentrations in the ACC before and after menopause. The relationship of the GABA levels with the patients’ scores on the 14-item Hamilton Anxiety Scale and 17-item Hamilton Depression Scale was determined.

**Results:**

GABA decreased with time. The postmenopausal GABA levels were significantly lower in the depression group than in the anxiety group and were significantly lower in both these groups than in the normal group. The postmenopausal GABA levels were significantly lower than the premenopausal levels in the normal, anxiety, and depression groups (*P* = 0.014, <0.001, and <0.001, respectively). The premenopausal GABA levels did not significantly differ between the normal vs. anxiety group (*P* = 0.907), normal vs. depression group (*P* = 0.495), and anxiety vs. depression group. The postmenopausal GABA levels were significantly lower in the depression group than in the anxiety group and were significantly lower in both these groups than in the normal group, normal vs. anxiety group (*P* = 0.022), normal vs. depression group (*P* < 0.001), and anxiety vs. depression group (*P* = 0.047).

**Conclusion:**

Changes in GABA concentrations in the anterior cingulate cortex are related with the pathophysiological mechanism and symptoms of perimenopausal depression.

## Introduction

The anterior cingulate cortex (ACC) is closely related to the occurrence and development of depression. The ACC occupies the rostral portions of Brodmann areas 24, 25, 32, and 33, and is activated by diverse tasks, ranging from emotion processing and regulation to attention and cognitive control (Ferrone et al., 2007). Many previous studies have confirmed the significant association of the ACC, especially the subgenual ACC, with the processing of negative emotions. The pregenual ACC is considered to be associated with cognitive functions such as social cognition, including theory of mind tasks and conflict monitoring (Ferrone et al., 2007; Formica et al., 2007; Ferolla et al., 2011).

Perimenopausal depression is a mental disorder that first occurs in women during the perimenopausal period and is mainly characterized by symptoms of hypothymia, anxiety, nervousness, and loss of interest, accompanied with autonomic and endocrine dysfunction, especially recession of the gonads. Women with severe symptoms may have a tendency to commit suicide. A meta-analysis has shown that women in the perimenopausal period were particularly vulnerable to anxiety or depression, and had more severe symptoms than women in the premenopausal period (de Kruif et al., 2016). However, the pathophysiological mechanisms of perimenopausal depression are still unknown.

Gamma-aminobutyric acid (GABA) is the main inhibitory neurotransmitter in the central nervous system. It combines with GABA receptors and inhibits excitatory neural activity. Abnormal GABA metabolism plays an important role in the pathogenesis of mental disorders such as depression and schizophrenia (Levy and Degnan, 2013; Rowland et al., 2013). An increasing body of preclinical and clinical evidence has proved that a close relationship exists between GABA and depression. Magnetic resonance spectroscopy (MRS) is a non-invasive technique for quantifying metabolites in the brain. MRS has been successfully applied in studies of depression and has detected changes in many metabolites in different brain regions (Puts and Edden, 2012). MRS plays an important role in exploring the treatments and mechanisms of depression. However, due to the chemical shift and the scalar coupling effect, the GABA spectrum overlaps with signals of other major metabolites. It is therefore difficult to detect GABA by using conventional ^1^H-MRS. An improved MRS method—MEGA-PRESS, based on partially refocused J-couplings—has been used to detect GABA in studies of healthy brains and psychiatric diseases.

In this study, we used the MEGA-PRESS technique to detect GABA in the anterior cingulate cortex (ACC) of perimenopausal women. The Totally automatic robust quantitation in nuclear MR (TARQUIN) software was used as the post-processing method to calculate GABA concentrations. We aimed to characterize the pathophysiological mechanisms of perimenopausal depression by determining whether changes in GABA concentrations in the ACC were associated with perimenopausal anxiety/depression.

## Materials and Methods

### Participants

**FIGURE 1:**
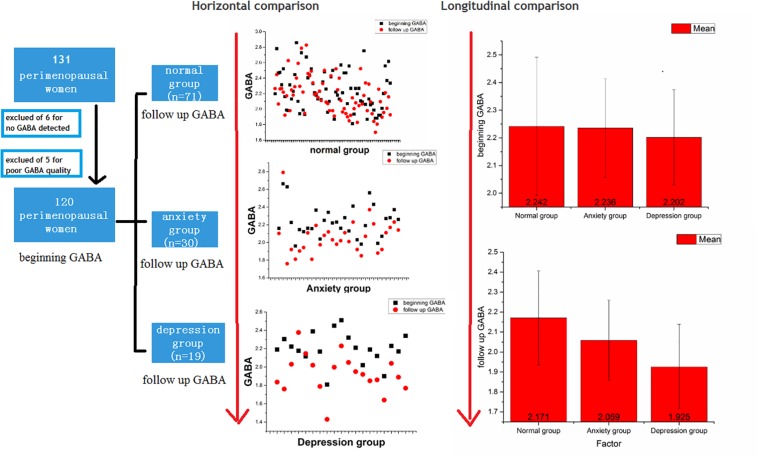
Flow chart and comparisons of GABA levels between the normal, anxiety, and depression groups.

We recruited 131 perimenopausal women. After the exclusion of 11 participants, 120 participants remained. The inclusion criteria for the experimental group were as follows: (1) women in the perimenopausal stage, as defined by the Stages of Reproductive Aging Workshop (Soules et al., 2001), i.e., a persistent ≥7-day difference in menstrual cycle length in consecutive cycles (persistence was defined as recurrence within 10 cycles of the first cycle with variable length), and (2) education up to junior high school level or above. The exclusion criteria were as follows: (1) GABA values could not be detected (6 of the 131 participants were excluded due to this reason), (2) diagnosis of a somatic disease (hypertension or diabetes mellitus); (3) presence of a hypothalamic-pituitary-adrenal axis or thyroid disease; (4) history of depression or other related mental illnesses, or presence of dementia or other organic mental disorders; (5) use of oral contraceptives or hormone therapy within 3 months of entering the study; (6) a history of non-depressive disorders in the participant or a family member (including immediate family by blood and collateral blood relatives within three generations); (7) smoking and/or dependence on alcohol; and (8) poor GABA quality (5 of the 131 participants were excluded because of this reason) (Figure 1).

#### Estrogen Measurements

A fasting blood specimen (3 mL) was collected into a 5-mL sterile plain tube without anticoagulant at 9:00–11:00 a.m. on the second or third day of the menstrual cycle. However, if a timely sample could not be obtained (as was the case for the late stage perimenopausal women), a fasting sample was taken when the endometrium was <5 mm thick as determined using transvaginal Doppler ultrasonography. In this study, estrogen levels fluctuated during the perimenopausal period, but the overall trend was downward.

This study was approved by the ethics committee of the Shanghai Sixth People’s Hospital, Shanghai Jiao Tong University), and all participants signed informed consent forms before being entered into the study.

### MRI and MRS Analyses

In all subjects, MR data were acquired using a 3.0-T MR scanner (MAGETOM, Verio, Siemens Healthcare, Erlangen, Germany) equipped with a 32-channel phased-array head coil as the transmitting and receiving coils. First, a T1-weighted turbo field echo sequence was used to obtain high-resolution three-dimensional (3D) axial images of the brain structure, with the following scanning parameters: field of vision (FOV), 230 mm; repetition time (TR)/echo time (TE), 1500/2.96 ms; flip angle, 9°; voxel size, 0.9 mm × 0.9 mm × 1 mm; slice thickness, 1 mm; and distance factor, 50%. The MEGA-PRESS sequence was used to detect GABA in the regions of interest (ROIs), with the following scanning parameters: TE, 68 ms; TR, 1500 ms; acquisition bandwidth, 1200 Hz; pulse placement, 1.9 ppm, and number of excitations, 64 on and 64 off. Unsuppressed water was used for water scaling and correction of frequency and phase. The spectra were fitted using TARQUIN software (Wilson et al., 2011; Mullins et al., 2014; Harris et al., 2017). GABA peaks were quantified calculated using the water-scaled method, as described previously (Wilson et al., 2011; Mullins et al., 2014; Harris et al., 2017).

**FIGURE 2:**
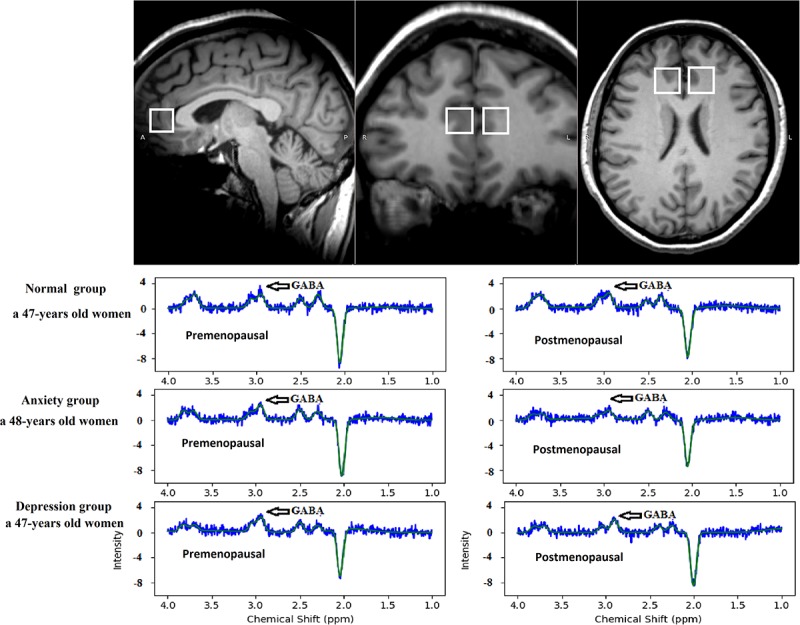
The region of interest in the anterior cingulate cortex on MEGA-PRESS in the axial, sagittal, and coronal planes. TARQUIN edited spectrum GABA in the normal, anxiety, and depression groups. The edited spectrum of the premenopausal and postmenopausal GABA levels located at 3.0 ppm.

A radiologist with 10 years of experience placed two ROIs measuring 2 cm × 2 cm × 2 cm each bilaterally in the subgenual ACC in the sagittal plane and adjusted them accordingly in the coronal and axial planes. The average GABA value of the right and left sides (ROIs) was calculated. The edges of all ROIs were positioned to avoid the lateral ventricles and skull. All images were post-processed by the same radiologist with 10 years of experience, and the TARQUIN software was used to calculate GABA concentrations in the ROIs (Figure 2).

Gray matter and white matter tissue proportion of interest has been evaluated for each patient using manual segmentation available in ITK-SNAP^1^, where the bounding box was redrawn from the saved planning volume of interest.

#### Quality Control

In order to gain a high success ratio acquiring GABA spectra, we defined a pre-requirement for performing GABA acquisition: magnetic field inhomogeneity <15 Hz for the defined ROI. After the GABA acquisition, we visually inspected the spectra, and the quality control parameters for spectral fitting were reviewed to verify that the spectra were not qualitatively abnormal. All full widths at half maximum were ≤0.12 ppm. Poorly fitted spectra higher than 20% of the GABA estimate were excluded from further analysis, and this led to the exclusion of 5 of the 131 participants.

### Statistical Analysis

**FIGURE 3:**
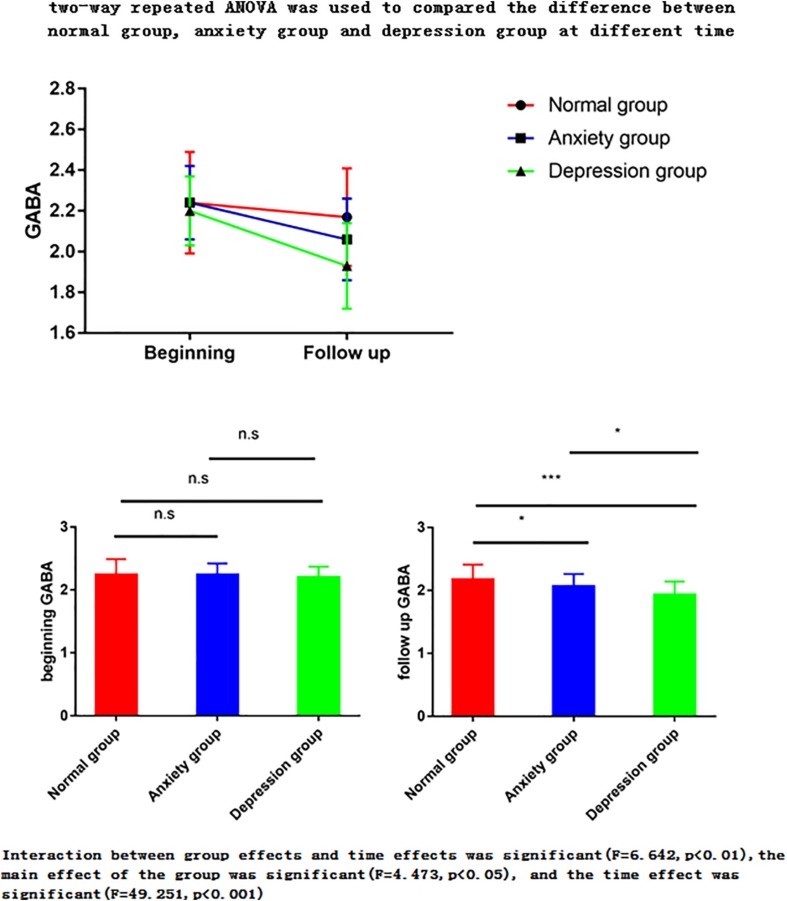
Two-way repeated ANOVA was used to compare the difference between normal group, anxiety group and depression group at different times. The ANOVA for repeated measures is used to perform the data analysis. Interaction between group effects and time effects was significant (*F* = 6.642, *p* < 0.01), the main effect of the group was significant (*F* = 4.473, *p* < 0.05), and the time effect was significant (*F* = 49.251, *p* < 0.001). (1) The main effect showed no statistical difference between any two groups in the three groups at the start time point, but there was a statistical difference between the two groups in the follow-up time point (*p* < 0.05). (2) The time effect shows that there are differences between the two time points in the normal group, there are differences between the two time points of the anxiety group, and there are also differences between the two time points in the depression group. ^∗^*P* < 0.05; ^∗∗^*P* < 0.01; ^∗∗∗^*P* < 0.001.

For all statistical tests, the level of significance was set at *P* < 0.05. The following analyses were carried out (Figure 3).

(1) Two-way repeated ANOVA with time (pre-menopausal, post-menopausal) and three groups (normal, depression, anxiety group) was used to compared changes GABA with time and groups. Both of the main effect of the group and the time effect were done.
  Main effect compared the difference between normal group, depression group and anxiety group at premenopausal stage (i.e., normal group vs. anxiety group, normal group vs. depression group, and anxiety group vs. depression group). It also compared the difference between the three groups at postmenopausal stage.
  Time effect was used to compare the difference of pre- and postmenopausal GABA concentrations in the normal group (*n* = 71), anxiety group (*n* = 30), and depression group (*n* = 19).
(2) The Pearson correlation coefficient was used to analyze the correlation of GABA with the Hamilton Anxiety Scale (HAMA)-14 scores in the anxiety group and with the Hamilton Depression Scale (HAMD)-17 scores in the depression group. A two-tailed test of significance was used, with a *P* value <0.05 considered statistically significant.
(3) Receiver operating characteristic (ROC) curves and the area under the curve (AUC) were calculated to evaluate the diagnostic performance of GABA concentration in the control, anxiety, and depression groups.
(4) Retrospective calculated the gray matter/white matter ratio, as this ratio changes at the two time points (beginning and follow up), one-way ANOVA was used to compare the difference.

## Results

### General Information

The 120 women remaining in this study were followed up for 18–24 months. All women underwent MRS with a 3.0-T MR scanner assessed before and after menopause. The participants were divided into three groups: normal group (*n* = 71), anxiety group (*n* = 30), and depression group (*n* = 19). These diagnoses were based on the Diagnostic and Statistical Manual of Mental Disorders, fifth edition (DSM-V) and were made by two psychiatrists (with 8 and 10 years of experience). These two psychiatrists also assessed the HAMA-14 and HAMD-17 scores before and after menopause. HAMD-17 scores ≥ 17 suggest depression, while HAMA-14 scores > 14 suggest anxiety (Figure 1).

The general information of the three groups is displayed in Table 1. Blood pressure, blood glucose and triglyceride levels, and body mass index did not significantly differ between the three study groups (*P* > 0.05; Table 1).

**TABLE 1:** General information.

	**Normal group**		**Anxiety group**		**Depression group**		***P***		
	**Mean**	***SD***	**Mean**	***SD***	**Mean**	***SD***	**P1**	**P2**	**P3**
Patients (n)	71		30		19		–	–	–
Age (years)	46.80	1.98	47.57	1.99	47.68	1.86	0.076	0.085	0.839
Systolic pressure (mmHg)	80.83	7.46	86.7	3.82	87.79	4.72	<0.05^∗^	<0.05^∗^	0.559
Diastolic pressure (mmHg)	122.96	7.96	125.83	6.16	126.74	6.73	0.075	0.049	0.676
Triglyceride (mmol/L)	1.414	0.306	1.551	0.237	1.452	0.274	0.030^∗^	0.612	0.238
BMI (kg/m^2^)	22.910	1.923	23.967	2.950	23.595	3.027	0.046	0.272	0.599
Premenopausal Serum estradiol (mmol/L)	83.170	31.204	81.142	33.245	92.588	30.500	0.769	0.251	0.219
Postmenopausal Serum estradiol (mmol/L)	75.858	17.334	63.478	22.195	54.020	18.483	0.003	<0.01^∗^	0.089
Premenopausal HAMD score					4.789	0.713	0.462	0.382	0.738
Postmenopausal HAMD score					20.737	2.579	<0.05^∗^	<0.05^∗^	<0.05^∗^
Premenopausal HAMA score			3.700	1.088			0.126	0.140	0.868
Postmenopausal HAMA score			18.1	3.356			<0.05^∗^	<0.05^∗^	<0.05^∗^

*P1, normal vs. anxiety group, independent-samples *t*-test; P2, normal vs. depression group, independent-samples *t*-test; P3, anxiety vs. depression group, independent-samples *t*-test. ^∗^*P* < 0.05 was statistically significant. BMI, body mass index; HAMD, hamilton depression scale; HAMA, hamilton anxiety scale.*

### MRS Data

By means of the MEGA-PRESS sequence, we successfully acquired edited spectra of GABA from the ACC region in 120 subjects.

#### GABA

The two-way repeated ANOVA results showed that the interaction between group effects and time effects was significant (*F* = 6.642, *p* < 0.01), the main effect of the group was significant (*F* = 4.473, *p* < 0.05), and the time effect was significant (*F* = 49.251, *p* < 0.001) (Tables 2–4).

**TABLE 2:** Two-way repeated ANOVA was used to compare the difference between normal group, anxiety group and depression group at different times.

**Group**	**Beginning GABA**	**Follow up GABA**	**F time**	**F group**	**F time × group**
Normal group	2.242 + 0.250	2.172 + 0.235	49.251^∗∗∗^	4.473^∗^	6.642^∗∗^
Anxiety group	2.236 + 0.180	2.059 + 0.201			
Depression group	2.202 + 0.172	1.923 + 0.213			

*Interaction between group effects and time effects was significant (*F* = 6.642, *p* < 0.01), the main effect of the group was significant (*F* = 4.473, *p* < 0.05), and the time effect was significant (*F* = 49.251, *p* < 0.001).^∗^*P* < 0.05; ^∗∗^*P* < 0.01; ^∗∗∗^*P* < 0.001.*

**TABLE 3:** Difference between normal group, depression group and anxiety group at premenopausal and postmenopausal time.

			**Mean Difference**	**Standard**		**95% Confidence interval**	
**Time**	**(I) Group**	**(J) Group**	**(I−J)**	**Error**	**Significant**	**for difference**	
						**Lower Bound**	**Upper Bound**
Beginning	Normal group	Anxiety group	0.006	0.049	0.907	–0.091	0.102
		Depression group	0.040	0.058	0.495	–0.075	0.154
	Anxiety group	Normal group	–0.006	0.049	0.907	–0.102	0.091
		Depression group	0.034	0.066	0.606	–0.096	0.164
	Depression group	Normal group	–0.040	0.058	0.495	–0.154	0.075
		Anxiety group	–0.034	0.066	0.606	–0.164	0.096
Follow up	Normal group	Anxiety group	0.113^∗^	0.049	0.022^∗^	0.017	0.210
		Depression group	0.245^∗^	0.058	0.000^∗∗∗^	0.131	0.360
	Anxiety group	Normal group	−0.113^∗^	0.049	0.022^∗^	–0.210	–0.017
		Depression group	0.132^∗^	0.066	0.047^∗^	0.002	0.262
	Depression group	Normal group	−0.245^∗^	0.058	0.000^∗∗∗^	–0.360	–0.131
		Anxiety group	−0.132^∗^	0.066	0.047^∗^	–0.262	–0.002

*The simple effect showed no statistical difference between any two groups in the three groups at the start time point, but there was a statistical difference between the two groups in the follow-up time point (*p* < 0.05). ^∗^*P* < 0.05; ^∗∗∗^*P* < 0.001.*

**TABLE 4:** Difference between premenopausal (time 1) and postmenopausal time (time 2) of the normal group, depression group and anxiety group.

			**Mean Difference**	**Std.**		**95% Confidence interval**	
**Group**	**(I) time**	**(J) time**	**(I-J)**	**Error**	**Sig.**	**for difference**	
						**Lower bound**	**Upper bound**
Normal group	1	2	0.070^∗^	0.028	0.014^∗^	0.015	0.125
	2	1	−0.070^∗^	0.028	0.014^∗^	–0.125	–0.015
Anxiety group	1	2	0.178^∗^	0.043	0.000^∗∗∗^	0.092	0.263
	2	1	−0.178^∗^	0.043	0.000^∗∗∗^	–0.263	–0.092
Depression group	1	2	0.276^∗^	0.054	0.000^∗∗∗^	0.169	0.383
	2	1	−0.276^∗^	0.054	0.000^∗∗∗^	–0.383	–0.169

*The simple effect shows that there are differences between the two time points in the normal group, there are differences between the two time points of the anxiety group, and there are also differences between the two time points in the depression group. ^∗^*P* < 0.05; ^∗∗∗^*P* < 0.001.*

(1) The main effect showed no statistical difference between any two groups in the three groups at the premenopausal time point, but there was a statistical difference between the two groups in the follow-up time point (*p* < 0.05).
  The postmenopausal GABA levels significantly differed between the normal vs. anxiety group (*P* = 0.022), normal vs. depression group (*P* < 0.001), and anxiety vs. depression group (*P* = 0.047). The postmenopausal GABA levels were significantly lower in the depression group than in the anxiety group and were significantly lower in both these groups than in the normal group.
  The premenopausal GABA levels did not significantly differ between the normal vs. anxiety group (*P* = 0.907), normal vs. depression group (*P* = 0.495), and anxiety vs. depression group (*P* = 0.606).
(2) The time effect shows that there are differences between the two time points in the normal group, there are differences between the two time points of the anxiety group, and there are also differences between the two time points in the depression group. This means there is difference between postmenopausal groups, and GABA decreased with time.
  The postmenopausal GABA levels were significantly lower than the premenopausal levels in the normal, anxiety, and depression groups (*P* = 0.014, <0.001, and <0.001, respectively).

#### Pearson Correlation Analysis

**FIGURE 4:**
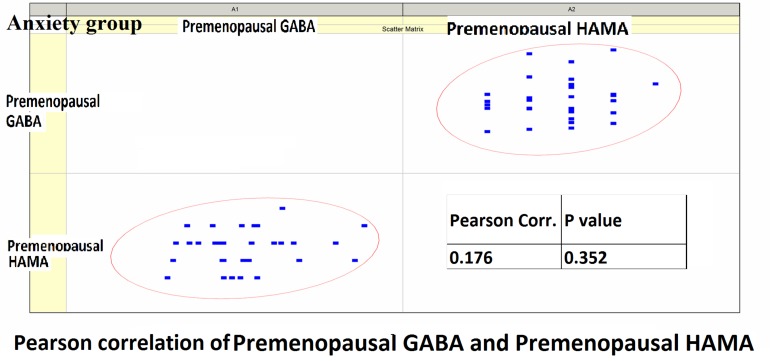
Pearson correlation coefficients for premenopausal GABA levels in the anxiety group and the premenopausal HAMA-14 scores.

**FIGURE 5:**
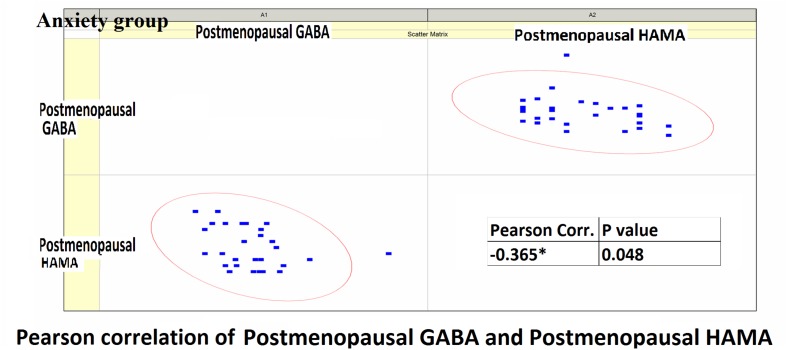
Pearson correlation coefficients for postmenopausal GABA levels in the anxiety group and the postmenopausal HAMA-14 scores.

**FIGURE 6:**
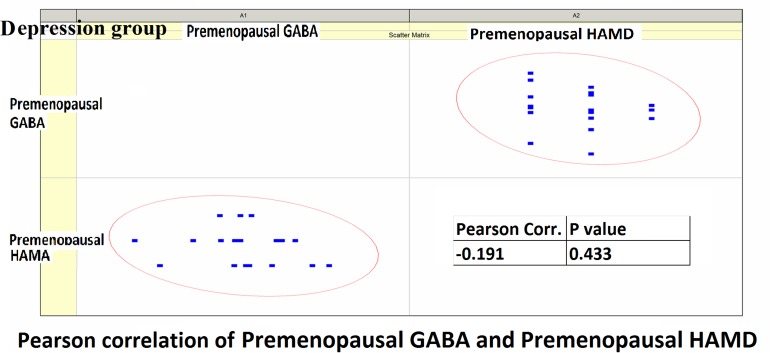
Pearson correlation coefficients for premenopausal GABA levels in the depression group and premenopausal HAMD-17 scores.

**FIGURE 7:**
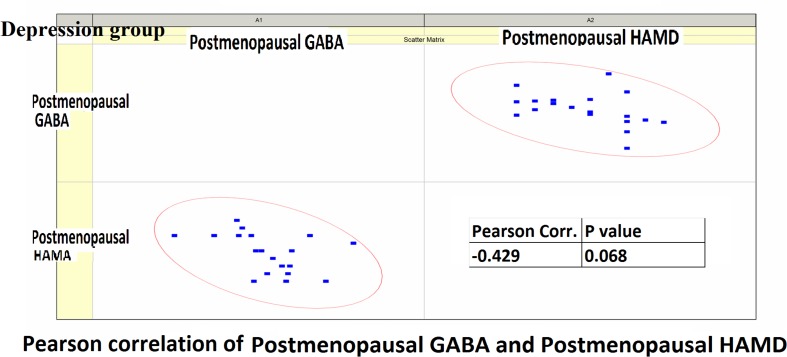
Pearson correlation coefficients for postmenopausal GABA levels in the depression group and postmenopausal HAMD-17 scores.

Pearson correlation analysis revealed the following: (1) The premenopausal GABA levels were not significantly correlated with the HAMA-14 scores in the anxiety group (*r* = 0.176, *P* = 0.352) or with HAMD-17 scores in the depression group (*r* = −0.191, *P* = 0.433). (2) The postmenopausal GABA levels were significantly correlated with the HAMA-14 score in the anxiety group (*r* = −0.365, *P* = 0.048), but not with the HAMD-17 score in the depression group (*r* = −0.428, *P* = 0.068; Figures 4–7 and Table 5).

**TABLE 5:** Pearson correlation coefficients for HAMA scores and GABA of anxiety group, Pearson correlation coefficients for HAMD scores and GABA of depression group.

	**Premenopausal**				**Postmenopausal**			
			**Pearson**				**Pearson**	
	**HAMA score**	**GABA**	**correlation**	***P* value**	**HAMA score**	**GABA**	**correlation**	***P* value**
Anxiety group	3.700 + 1.088	2.236 + 0.180	0.176	0.352	18.1 + 3.356	2.059 + 0.201	−0.365^∗^	0.048

	**Premenopausal**				**Postmenopausal**			
	**HAMD score**	**GABA**			**HAMD score**	**GABA**		

Depression group	4.789 + 0.713	2.202 + 0.172	−0.191	0.433	20.737 + 2.579	1.923 + 0.213	−0.42857	0.068

*Two-tailed Pearson correlation test of significance was used. ^∗^Correlations were significant at the 0.05 probability level.*

**FIGURE 8:**
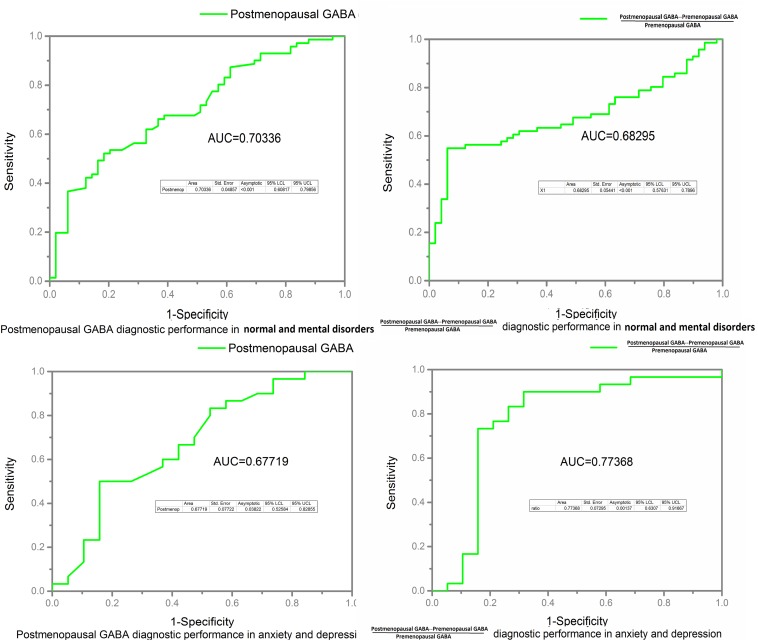
Receiver operating characteristic (ROC) curves and area under the curve (AUC) value were calculated to assess the diagnostic performance of GABA in the normal, anxiety, and depression groups.

Receiver operating characteristic curve and AUC results: The postmenopausal GABA diagnostic performance in normal and mental disorders (both anxiety and depression) was as follows: AUC value 0.703 (*P* < 0.001). Postmenopausal GABA diagnostic performance in anxiety and depression was as follows: AUC value 0.677 (*P* = 0.038). The ratio of GABA decline to premenopausal GABA diagnostic performance in normal and mental disorders (both anxiety and depression) was as follows: AUC value 0.683 (*P* < 0.001). The ratio of GABA decline to premenopausal GABA diagnostic performance in anxiety and depression was as follows: AUC value 0.774 (*P* = 0.001; Figure 8).

#### Gray Matter/White Matter Ratio

There is no significant difference between groups and times; gray matter/white matter ratio did not drive the GABA comparison results (Table 6).

**TABLE 6:** Gray matter/white matter ratio comparison between three groups (normal group, anxiety group, and depression group) at beginning time and follow up time.

		**Number**	**Lesion**		**Comparison between groups**		
			**Mean**	***SD***		***T* value**	***P* value**
Beginning	Normal group	71	0.774	0.013	Anxiety group Normal group	–0.176	0.860
	Anxiety group	30	0.773	0.013	Depression group Normal group	1.686	0.094
	Depression group	19	0.779	0.015	Depression group Anxiety group	1.616	0.109
Follow up	Normal group	71	0.778	0.019	Anxiety group Normal group	–0.929	0.355
	Anxiety group	30	0.774	0.017	Depression group Normal group	0.686	0.494
	Depression group	19	0.774	0.019	Depression group Anxiety group	0.086	0.932

*There is no significant difference between groups and times of Gray matter/white matter ratio.*

GABA SNR%SD were calculated between groups and two time points, there is no significant difference.

## Discussion

In this study, we have provided preliminary evidence that GABA levels in the ACC region of perimenopausal women with depression were significantly lower after menopause and were significantly lower than the levels in the control group (*P* < 0.05). This finding suggests that a reduction in GABA levels in the ACC is associated with the pathophysiological mechanism of perimenopausal depression, and that there might be a lack of GABA in the perimenopausal period. The study also found that after menopause, the concentration of GABA was significantly lower in the depression group than in the anxiety group, and significantly lower in the anxiety group than in the normal group. This further suggested an association between a reduction in GABA levels and depression. We also observed dynamic changes in GABA levels during the transition from the premenopausal to the postmenopausal period.

Many investigators have focused on the relationship between GABA levels and depression. GABA concentrations in the plasma and cerebrospinal fluid of depressive patients are lower than those in healthy controls. Smiley et al. (2016) found that GABA-neuron density in the auditory cerebral cortex is reduced in subjects with major depressive disorder. Using MRS studies, Hasler et al. (2007) found lower GABA levels in the prefrontal cortex in subjects with major depression than in healthy controls. Many studies have found that patients with depression have lower GABA levels in the occipital lobe and ACC than do healthy controls (Sanacora et al., 2004; Bhagwagar et al., 2008). Sanacora et al. (2004) have suggested that GABA concentrations vary among the different subtypes of depression and that a change in the ratio of excitatory–inhibitory neurotransmitter levels might be associated with abnormal brain function. In investigations of female physiological cycles related with depression, Liu et al. (2015) found that GABA levels in the ACC, prefrontal lobe, and left basal ganglia region were significantly reduced in women with premenstrual dysphoric disorder. Wang et al. (2016) found that GABA levels in the ACC and medial prefrontal lobe were decreased in postmenopausal women. In our study, we also detected reduced GABA levels in the ACC of perimenopausal women with depression, which was consistent with the results of the above studies. However, GABA was too low to be detected in some participants.

An imbalance of the limbic-cortical-striatal-pallidal-thalamic loop is the generally acknowledged neurological model of depression. The activity of the anterior cingulate gyrus and dorsal lateral frontal lobe has been shown to be decreased in depressive patients. Wang et al. (2016) detected significantly low GABA levels in the ACC/medial prefrontal cortex of postmenopausal women with depression. Dubin et al. (2016) found that after repetitive transcranial magnetic stimulation, the GABA levels in the medial prefrontal cortex significantly increased. Therefore, in this study, we selected the ACC region as the ROI, as this region is closely related with emotional function. We did not choose the occipital lobe as in the study by Bhagwagar et al. (2008) because the size of the ROI in our study was relatively large, and ROIs placed in the occipital lobe can be easily disturbed by other structural signals in the base of the skull. Hasler et al. (2007) detected reduced GABA levels in the prefrontal regions in patients with major depressive disorder, including parts of the ACC, but the GABA values in their study were lower than the GABA values in this study, possibly because of differences in ROI sizes, channels of the head coil, and calculation methods. We retrospectively calculated the gray matter/white matter ratio, as this ratio changes at the two time points (beginning and follow up); there was no significant difference between groups and times. Gray matter/white matter ratio did not drive the GABA results.

There are some limitations to this study. First, this is a cohort study, and the sample size needs to be increased in future. The follow-up period was relatively short. To explain the relationship between GABA levels and the pathogenesis of perimenopausal depression, more experiments need to be performed. GABA spectroscopy was poor due to movement, and GABA spectroscopy could not be acquired due to the participant requesting to end the scan prematurely. Furthermore, we only selected the ACC as the ROI; the prefrontal cortex and other brain regions related to emotional circuits were not included in this study. The differences between the left and right ROIs were not assessed. The manually prescribed ROIs were subjective and could have led to deviations in the results of different subjects. Moreover, it has been suggested that the GABA levels in the brain decrease with age (Gao et al., 2013). During the perimenopausal period, hormone levels fluctuate significantly. Studies have suggested that decreased estrogen levels affect perimenopausal depression (Schmidt et al., 1994). And the gray matter/white matter ratio was retrospectively calculated, and a small amount of cerebrospinal fluid was not considered and may lead to some small deviation.

## Conclusion

In summary, this study examined the changes in the GABA levels in the ACC region in perimenopausal women with depression and anxiety as well as healthy women (controls). The results suggested that the GABA levels in the ACC decreased during the perimenopausal period, and that this decrease was closely associated with depression and anxiety. We calculated GABA concentrations by using the MEGA-PRESS sequence; the results were highly reliable and stable. Advances in MRS technology will be important in the exploration of pathogenesis and the development of targeted drugs for perimenopausal depression.

## Ethics Statement

This study was approved by the local ethics committee (The ethics committee of Shanghai Sixth Affiliated People’s Hospital, Shanghai Jiao Tong University), and all participants signed informed consent forms before entering the study.

## Author Contributions

DW wrote the manuscript. DW, XW, and M-TL calculated the statistical data. Y-HL and HW designed the research content and research direction.

## Conflict of Interest Statement

The authors declare that the research was conducted in the absence of any commercial or financial relationships that could be construed as a potential conflict of interest.

## Footnotes

1 http://www.itksnap.org
